# IgA Vasculitis Nephritis: A Case Series and Comparison of Treatment Guidelines

**DOI:** 10.1155/2020/8863858

**Published:** 2020-12-02

**Authors:** Abdalhamid Al Harash, Stephanie Saeli, Michael Lucke, Swati Arora

**Affiliations:** ^1^Division of Rheumatology, Department of Medicine, Allegheny Health Network, Pittsburgh, PA, USA; ^2^Department of Medicine, Allegheny Health Network, Pittsburgh, PA, USA; ^3^Division of Nephrology, Department of Medicine, Allegheny Health Network, Pittsburgh, PA, USA

## Abstract

Immunoglobulin A (IgA) vasculitis nephritis (IgAVN) and IgA nephropathy (IgAN) share many pathological parallels and are viewed as related diseases by many groups. Current treatment guidelines remain vague, controversial, and without consensus, especially regarding the role of immunosuppressive medications. We present five cases of IgAVN encountered at our tertiary care center between 2016 and 2020, which were treated with different immunosuppression regimens. Infection was the leading cause of death in this series. These cases provide evidence that IgAVN should be distinguished from IgAN on a spectrum of IgA-associated glomerulonephritis. The outcomes presented herein suggest that the morbidity and systematic involvement IgAVN is greater than previously believed and that these substantial risks should be reflected in contemporary treatment guidelines.

## 1. Introduction

IgA vasculitis is a systemic disease that impacts small blood vessels and is characterized by the IgA deposits in the kidneys, skin, and other organs [1]. IgAVN and IgAN share many clinicopathologic features and are considered related diseases [2]. Existing treatment guidelines remain vague and controversial, especially regarding the role of immunosuppressive medications. Kidney Disease Improving Global Outcomes (KDIGO) recommend a similar treatment approach for both IgAN and IgAVN, grouping them in the same disease category [3]. On the contrary, European treatment guidelines view IgAN and IgAVN as two distinct entities; however, these clinical references are for pediatric population and cannot always be extrapolated to an adult population [4]. Both KDIGO and European guidelines recommend a treatment strategy of cyclophosphamide (CYC) and immunosuppression with steroids for severe disease; however, there is no consensus on the use of immunosuppressive drugs for mild to moderate disease. Finally, IgAN and IgAVN have relatively low incidence, making randomized controlled studies that might inform therapy guidelines difficult to conduct.

We present five cases of IgAVN encountered at our tertiary care center between 2016 and 2020 treated with aggressive immunosuppression (Table 1). This series demonstrates that extrapolating IgAN treatment guidelines to cases of IgAVN is insufficient, as evidence suggests that these diseases, though related, diverge in a number of ways and their clinical associations should be distinguished from one another. We argue that IgAVN is more morbid and systemic than its counterpart, and accordingly, a more aggressive therapy with immunosuppression should be administered even for moderate nephritis.

### 1.1. Case 1

A 22-year-old male presented with a papulopustular rash over the bilateral lower extremities, left elbow synovitis, and generalized abdominal tenderness. He reported a sore throat and fever the week before that had since subsided. The serum creatinine level was 0.88 mg/dL. He was found to have small bowel enteritis on CT scan. Skin biopsy showed leukocytoclastic vasculitis with IgA deposits on immunofluorescence (IF). He was started on IV methylprednisolone due to worsening abdominal and joint pain. After a dramatic improvement in his symptoms, he was discharged from the hospital on an oral glucocorticoid taper.

**Figure 1:**
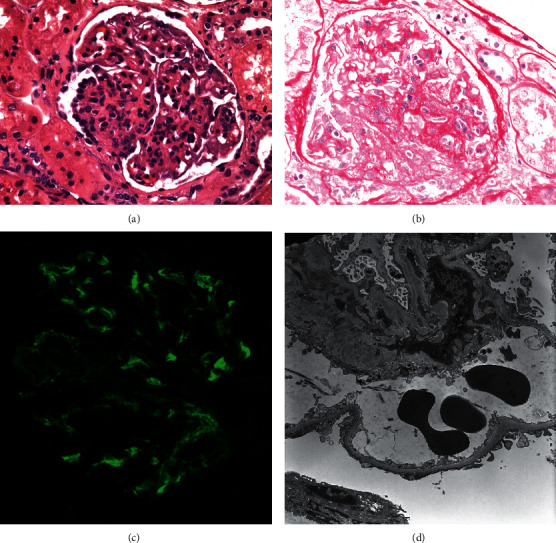
Light and electron microscopy, kidney biopsy from patient 1. (a) Hematoxylin and eosin stain showing endocapillary segmental hypercellularity. (b) PAS stain showing crescent formation. (c) DIF showing granular mesangial IgA staining (+2). (d) Electron microscopy showing segmental mesangial and paramesangial immune deposits. DIF: direct immunofluorescence. IgA: immunoglobulin A. PAS: periodic acid-schiff.

One week after cessation of steroid regimen, the patient developed gross hematuria. Urine protein-to-creatinine ratio (UPCR) was 5.75 g/day. His serum creatinine remained stable at 0.72 mg/dL. He was started on lisinopril and prednisone. Kidney biopsy was performed; this included 13 glomeruli, 2 of which showed focal cellular crescents. IF was positive for granular mesangial deposits of IgA, and electron microscopy revealed mesangial immune deposits (Figure 1). The patient was diagnosed with IgAVN and prednisone was resumed. While taking 60 mg prednisone, the patient developed acne and insomnia and was subsequently started on mycophenolate mofetil (MMF) titrated to 3 g daily. Prednisone was tapered over six weeks. Proteinuria improved and UPCR decreased to 0.22 g/day after 2 months. Rash showed improvement and neither joint nor abdominal symptoms returned. On five months follow-up, his proteinuria was 0.3 g/day. Serum creatinine remained stable.

### 1.2. Case 2

A 55-year-old male presented to the hospital with a 2-week history of petechial rash on the bilateral lower extremities. He denied arthralgia, abdominal pain or evidence of infection. He had a medical history of cerebrovascular accident 3 years prior to presentation, insulin-dependent diabetes mellitus, nonischemic cardiomyopathy, and heart failure with reduced ejection fraction. Palpable purpura was noted with otherwise unremarkable physical examination.

The creatinine level was 1.6 mg/dL, urinalysis showed microscopic hematuria with many red blood cell casts. The UPCR was 2 g/day. Skin biopsy showed leukocytoclastic vasculitis with evidence of IgA deposits on IF. On kidney biopsy, 15 glomeruli were noted, all showing a mild increase in mesangial matrix. In addition, up to 10 glomeruli showed mild segmental mesangial hypercellularity. No endocapillary proliferation, sclerosing lesions, or crescents were seen. IF was positive for IgA mesangial deposition. His presentation was consistent with IgAVN. The patient was treated with 3 days of pulse IV steroids then was started on an oral prednisone taper. Lisinopril was initiated, after which UPCR improved to 0.3 g/day and creatinine improved to 1 mg/dL.

Four months later, the patient presented to the hospital with influenza-B infection, multilobar pneumonia, and acute lacunar infarcts. His prednisone dosage was 30 mg daily at that time. He was treated and discharged. The patient passed away one day later at home. No autopsy was performed.

### 1.3. Case 3

A 42-year-old male presented to the hospital with lower extremity rash and abdominal pain. He was diagnosed with IgA vasculitis with skin involvement 13 years prior to presentation and treated with systemic glucocorticoids, which were tapered off. His medical history included diabetes, hypertension, and neuropathy. Physical examination was notable for palpable purpura in the lower extremities. Skin biopsy showed leukocytoclastic vasculitis with IgA deposits on IF. Serum creatinine level was normal. Urinalysis was not carried out at that time. He was discharged on 60 mg prednisone.

Four weeks later, the patient presented to the hospital with gross hematuria and edema of the lower extremities. His creatinine level was 4.5 mg/dL. Urinalysis was notable for many red blood cells and red blood cell casts. UPCR was 2.2 g/day. Kidney biopsy showed up to 13 glomeruli, 2 of which showed cellular crescent formations. There was no mesangial hypercellularity or endocapillary proliferation. Prominent mesangial IgA deposits were noted on IF.

One gram of IV methylprednisolone was given for 3 consecutive days, along with 400 mg/meter square of IV CYC once. Hemodialysis was administered due to refractory hyperkalemia. The patient was discharged on prednisone taper over 3 months. He was given 5 monthly doses of IV CYC as an outpatient (400 mg/meter square each). UPCR improved to 0.13 g/day and creatinine improved to 2.4 mg/dL. He remained off hemodialysis.

### 1.4. Case 4

A 45-year-old female presented to the hospital reporting rash and edema of the lower extremities for 2-weeks. She had upper respiratory infection symptoms 4 weeks prior to presentation. Her medical history was notable for stage 3 chronic kidney disease (baseline creatinine 1.2 mg/dL), membranoproliferative glomerulonephritis (previously treated with steroids and IV CYC), and Raynaud's and Sjogren's syndrome. She was on chronic MMF.

Physical exam was notable for purpura and edema of the bilateral lower extremities. The level of creatinine was 2.3 mg/dL, urinalysis showed many red blood cells, and UPCR was 6 g/day. Acute dyspnea developed and bronchoscopy revealed diffuse alveolar hemorrhage (DAH). Skin biopsy showed necrotizing vasculitis with IgA deposits. Renal biopsy showed diffuse immune complex membranoproliferative glomerulonephritis with prominent mesangial IgA deposition.

One gram IV methylprednisolone was given for a total of 3 g. Plasma exchange was started for a total of 5 sessions. Two doses of rituximab 1 g IV was given two weeks apart. MMF was stopped. Four weeks later, the patient presented to the hospital with fever and was found to be in septic shock due to Pseudomonas bacteremia. She developed purpura fulminans with worsening renal function, requiring initiation of hemodialysis. The patient ultimately progressed to multiorgan failure and expired.

### 1.5. Case 5

A 65-year-old female presented with petechial rash over her upper and lower extremities that developed 4 days after the onset of diarrheal illness. Skin biopsy showed leukocytoclastic vasculitis with IgA deposits on IF. The creatinine level was 1.09 mg/dL. She was treated with taper dose prednisone, which led to an improvement of her skin rash. However, one week later, while on 10 mg prednisone daily, the patient noticed foamy urine. The UPCR was 6 g/day and the creatinine was 1.01 mg/dL. Kidney biopsy showed focal segmental endocapillary hypercellularity and focal cellular crescents (2/12 glomeruli). IF was positive for mesangial IgA staining. Prednisone was increased to 1 mg/kg/day for two weeks followed by taper over 10 weeks to 10 mg per day with low dose lisinopril. An MMF regimen of 2 g daily was also added. Kidney function remained stable and the UPCR improved to 1.9 g/day over one month.

## 2. Discussion

The international consensus conference on nomenclature of systemic vasculitides defines IgA vasculitis (IgAV), also known as Henoch–Schönlein purpura (HSP), in the following terms: “a vasculitis with IgA-dominant immune deposits affecting small vessels and typically involving skin, gut, and glomeruli and associated with arthralgia or arthritis” [5]. When the disease affects the kidneys, this condition is called Immunoglobulin A (IgA) vasculitis nephritis (IgAVN). Nearly two centuries after HSP was described, Berger and Hinglais first reported a form of glomerulonephritis associated with mesangial accumulation of IgA with focal and segmental mesangial proliferation, a condition later termed IgA nephropathy (IgAN) [6].

KDIGO [3] advises similar treatment strategies for both IgAN and IgAVN. These guidelines recommend early treatment with renin-angiotensin inhibitor and subsequent initiation of steroids if proteinuria >1 g/day persists after 3–6 months of conservative management. They also advise against the use of CYC, azathioprine (AZA), or MMF for moderate nephritis. Of note, because these guidelines are extrapolated from IgAN treatment strategies, it is our belief that they may be of limited utility for some cases of IgAVN. European guidelines, on the contrary, consider IgAN and IgAVN two distinct entities and suggest oral steroids as first line therapy, with MMF or AZA as first- or second-line therapy. However, it is important to note that these recommendations were designed for a pediatric population [4].

Treatment recommendations for IgAVN differ with regard to the severity of the disease. Mild disease is defined as a mild to moderate proteinuria (mild proteinuria, <1 g/day; moderate proteinuria, <2.5 g/day) with normal glomerular filtration rate (GFR). Moderate disease is defined as <50% crescents on biopsy and impaired GFR or severe persistent proteinuria (>2.5 g/day for 4 weeks). Severe disease is defined by the presence of >50% crescents and impaired GFR or severe persistent proteinuria [4]. DAH, though rare, calls for aggressive immunosuppression; similar to the management of DAH induced by other vasculitic causes. Both KDIGO and European guidelines recommend immunosuppression with steroids along with CYC for severe IgAVN [3, 4]. However, in patients with mild to moderate disease, there is no clear consensus on immunosuppression.

Only one patient in this series presented with the disorder's archetypal triad of arthritis, enteritis, and purpura. Another patient presented with DAH, a rare manifestation of IgAVN. All five patients underwent kidney and skin biopsies. While the addition of a skin biopsy is not standard in the diagnostic workup, it may help exclude other diagnoses in cases of atypical rashes with systemic involvement. The presence of IgA staining can also aid the diagnosis of IgAVN; however, absence of IgA does not exclude IgAVN [7].

In light of limited treatment guidelines, treating physicians in this series administered an aggressive course of therapy with the addition of immunosuppressive agents for all five patients. This strategy was met with some success. Each regimen was based on the clinical discretion of treating physicians and differed due to the heterogeneous presentation of the disease with varying severity. Patient 1 received steroids in a quick taper and MMF early in the course of disease. Proteinuria improved significantly and side effects from steroids were limited due to the short and low dose of prednisone used. On the contrary, patient 2 received steroid monotherapy, with high dose and prolonged course, but led to serious infection and death. Patient 3 received CYC early on in the course of therapy with taper dose steroids due to the findings of severe renal dysfunction and rapidly progressive glomerulonephritis. Patient 4, on the contrary, presented with a rare manifestation of IgAV with DAH and was treated with steroids and rituximab, which resulted in serious infection, DIC, and death. Patient 5 was treated with rapid taper of steroids with moderate dose MMF instituted from the start yielding a successful outcome.

All patients in this series had renal involvement with development of moderate-to severe proteinuria and/or renal dysfunction, prompting the performance of kidney biopsy. These outcomes are perhaps unsurprising considering the incidence of concomitant ESRD in adults with IgAVN is 11% [8]. Among IgAVN patients, renal involvement generally develops several days to one month after initial presentation. In a retrospective study of 260 adults with IgAVN, 30% of patients developed renal insufficiency and 88% showed hematuria and median proteinuria of 1.5 g/day [9].

IgAN and IgAVN share many pathological features, and the risk of progression to end-stage renal disease appears to be the same after 10 years [10]. Nevertheless, we argue that IgAN and IgAVN are exclusive diseases each warranting their own treatment guidelines. Both diseases have predominant IgA deposits in the mesangium and glomeruli; in IgAVN, however, capillary wall staining for IgA is more frequently found and is potentially more evident than mesangial deposition [11]. In contrast to IgAN, IgAVN is rapidly progressive and associated with underlying systemic vasculitis, multiorgan involvement, and endothelial injury. A history of preceding or concomitant infection is common. Additionally, IgAVN is primarily a childhood disease, whereas IgAN reaches peak incidence in the second or third decade of life. Although steroids are currently a first-line therapy for IgAN and IgAVN, it is important to note that these agents have been associated with serious and potentially fatal side effects. Recent trials have shown that utilizing high dose steroids in IgAN was associated with many adverse effects, including infection, weight gain, and impaired glucose. However, because we argue that patients with IgAVN comprise an altogether different patient population, such studies are an imperfect proxy for clinical scenarios involving IgAVN, and finding viable steroid-sparing therapies warrants continuing investigation. Recent evidence showed that when MMF with low dose steroids was compared to high dose taper prednisone, it was not proven to reduce proteinuria in patients with IgAN or IgAVN more effectively than steroids; critically, however, the MMF arm suffered from fewer side effects [12, 13]. Our series evidence further supported the notion that steroid-sparing drugs in moderate disease (MMF in patient 1 and 5) might reduce the side effects of steroids in higher risk populations, such as patients with diabetes. Other therapies are also promising. For instance, a small case series showed the benefit of B-cell depleting therapies along with steroids in patients with IgAVN or severe IgA nephropathy [14, 15].

## 3. Conclusion

Although IgAN and IgAVN share many pathological features, we argue that they are exclusive diseases each warranting their own treatment guidelines, particularly for an adult population. In our opinion, IgAVN requires aggressive immunosuppression therapy due to its systemic nature and high morbidity. Further studies are needed to establish safe and effective immunosuppression regimens.

## Figures and Tables

**Table 1:** IgAVN cases.

Patient	Age/race/gender	Organ involvement	Biopsy results	Therapy	Outcomes
1	22/C/M	Skin-abdomen-joints-kidney	<25% cellular crescents	GC, MMF	Improving proteinuria, in remission
2	55/C/M	Skin-kidney	Mesangial proliferation with IgA deposition	GC	Infection, death
3	42/C/M	Skin-kidney-abdomen	<25% cellular crescent	GC, CYC	Improving proteinuria, in remission
4	45/C/F	Skin-kidney-lung	MPGN with IgA-immune complex	GC, RTX	Infection, death
5	65/C/F	Skin-abdomen-kidney	<25% cellular crescents	GC, MMF	Improving proteinuria, stable creatinine

GC = glucocorticoids; MMF = mycophenolate mofetil; CYC = cyclophosphamide; RTX = rituximab; C = caucasian; F/M = female/male.

## Data Availability

Data used to support the findings of the study are available from the corresponding author upon request, after removing the patient identifiers.

## References

[1] Sepahi M. A., Mubarak M., Kellner J. S. (2020). Predicting renal outcomes in immunoglobulin A vasculitis nephritis; from ISKDC classification to oxford MEST-C classification. *Journal of Renal Injury Prevention*.

[2] Nicoara O., Twombley K. (2019). Immunoglobulin a nephropathy and immunoglobulin a vasculitis. *Pediatric Clinics of North America*.

[3] Cattran D. C., Feehally J., Terence Cook H. (2012). Kidney disease: improving global outcomes (KDIGO) glomerulonephritis work group. KDIGO clinical practice guideline for glomerulonephritis. *Kidney International Supplements*.

[4] Ozen S., Marks S. D., Brogan P. (2019). European consensus-based recommendations for diagnosis and treatment of immunoglobulin A vasculitis—the SHARE initiative. *Rheumatology*.

[5] Jennette J. C., Falk R. J., Andrassy K. (1994). Nomenclature of systemic vasculitides. Proposal of an international consensus conference. *Arthritis & Rheumatism*.

[6] Berger J., Hinglais N. (1968). Les depots intercapillares d’IgA-IgG. *Journal of Urology & Nephrology*.

[7] Larson A. R., Granter S. R. (2014). Utility of immunofluorescence testing for vascular IgA in adult patients with leukocytoclastic vasculitis. *American Journal of Clinical Pathology*.

[8] Pillebout E., Thervet E., Hill G., Alberti C., Vanhille P., Nochy D. (2002). Henoch–Schönlein purpura in adults: outcome and prognostic factors. *Journal of the American Society of Nephrology*.

[9] Audemard-Verger A., Terrier B., Dechartres A. (2017). Characteristics and management of IgA vasculitis (Henoch-Schönlein) in adults: data from 260 patients included in a French multicenter retrospective survey. *Arthritis & Rheumatology*.

[10] Davin J.-C., Ten Berge I. J., Weening J. J. (2001). What is the difference between IgA nephropathy and Henoch-Schönlein purpura nephritis?. *Kidney International*.

[11] Emancipator S. N., London H. R. H. (1993). Primary and secondary forms of IgA nephritis and Schönlein–Henoch syndrome. *Pathology of the Kidney*.

[12] Hou J.-H., Le W.-B., Chen N. (2017). Mycophenolate mofetil combined with prednisone versus full-dose prednisone in IgA nephropathy with active proliferative lesions: a randomized controlled trial. *American Journal of Kidney Diseases*.

[13] Ren P., Han F., Chen L. (2012). The combination of mycophenolate mofetil with corticosteroids induces remission of Henoch-Schönlein purpura nephritis. *American Journal of Nephrology*.

[14] Lundberg S., Westergren E., Smolander J., Bruchfeld A. (2017). B cell-depleting therapy with rituximab or of a tumumab in immunoglobulin A nephropathy or vasculitis with nephritis. *Clinical Kidney Journal*.

[15] Fenoglio R., Naretto C., Basolo B. (2017). Rituximab therapy for IgA-vasculitis with nephritis: a case series and review of the literature. *Immunologic Research*.

